# Mental health impacts of racial discrimination in Australian culturally and linguistically diverse communities: a cross-sectional survey

**DOI:** 10.1186/s12889-015-1661-1

**Published:** 2015-04-18

**Authors:** Angeline S Ferdinand, Yin Paradies, Margaret Kelaher

**Affiliations:** Centre for Health Policy, Melbourne School of Population and Global Health, University of Melbourne, Melbourne, Australia; Alfred Deakin Institute for Citizenship and Globalisation, Faculty of Arts and Education, Deakin University, Burwood, Australia

## Abstract

**Background:**

Racial discrimination denies those from racial and ethnic minority backgrounds access to rights such as the ability to participate equally and freely in community and public life, equitable service provision and freedom from violence. Our study was designed to examine how people from racial and ethnic minority backgrounds in four Australian localities experience and respond to racial discrimination, as well as associated health impacts.

**Methods:**

Data were collected from 1,139 Australians regarding types of racial discrimination experienced, settings for these incidents, response mechanisms and psychological distress as measured by the Kessler 6 (K6) Psychological Distress Scale.

**Results:**

Age, education, religion, gender, visibility and rurality were all significantly associated with differences in the frequency of experiencing racial discrimination. Experiencing racial discrimination was associated with worse mental health. Mental health impacts were not associated with the type of discriminatory experience, but experiencing racial discrimination in shops and in employment and government settings was associated with being above the threshold for high or very high psychological distress. One out of twelve response mechanisms was found to be associated with lower stress following a discriminatory incident.

**Conclusions:**

Study results indicate that poorer mental health was associated with the volume of discrimination experienced, rather than the type of experience. However, the impact of experiencing discrimination in some settings was shown to be particularly associated with high or very high psychological distress.

Our findings suggest that interventions designed to prevent the occurrence of racism have more potential to increase mental health in racial and ethnic minority communities than interventions that work with individuals in response to experiencing racism.

## Background

Discrimination describes a range of behaviours and practices that result in unfair and avoidable inequalities in power, resources and opportunities across groups [1] in society and serve to support systems of privilege and oppression [2]. Discrimination manifests across a continuum, from violence or illegal actions to subtle forms of social exclusion.

Racism can be broadly defined as the types of behaviours, practices, beliefs and prejudices that underlie systemic and avoidable inequalities in social power and opportunities across groups in society based on race, ethnicity, culture or religion. ‘Race’ refers to a cultural, rather than biological, construction of identity based on phenotypic expression, shared ancestry and/or cultural heritage [3,4]. Racism can occur at three conceptual levels, which overlap in practice: interpersonal racism (i.e., racist interactions between people); internalised racism (i.e., the incorporation of ideologies within the worldview of an individual who experiences racism which results in the unequal distribution of power between racial, ethnic, cultural or religious groups); and systemic or institutional racism (i.e., formal policies, practices, processes and conditions that serve to increase power differentials between racial, ethnic, cultural or religious groups) [4,5]. Racial discrimination is the expression of racism through actions taken at individual or institutional levels that lead to inequities across different racial, ethnic, cultural and/or religious groups. These definitions of racism and racial discrimination acknowledge the overlapping and intertwined spheres of racial, ethnic, cultural and religious identity both in social theory and in the lived experience of individuals and groups who experience racism and racial discrimination [6,7].

Exposure to racial discrimination is widely understood as a social determinant of health and a contributing factor to health inequities between racial and ethnic groups. The literature reports wide-ranging negative health outcomes for populations affected by racial discrimination. Physical health outcomes include increased prevalence of diabetes and cardiovascular effects as well as an increase in behaviours that would be expected to have a negative effect on health, such as smoking cigarettes and the use/misuse of alcohol and other drugs. In terms of mental health effects of racial discrimination, studies have noted that targets are at increased risk of developing a range of mental health problems such as anxiety and depression. In some studies, a dose–response effect has been noted, with individuals who report higher levels or more severe forms of racial discrimination experiencing higher risk of poor health than those who experience discrimination less frequently [8-11].

These impacts on health outcomes are understood to occur through a number of different pathways. Experiencing racial discrimination may result in individuals having restricted access to resources required for health, such as adequate housing, education and service provision. Stress and negative emotions resulting from exclusion may have detrimental psychological and physiological effects or lead targets of discrimination to pursue negative health behaviours such as alcohol or drug use as coping measures [11-13]. At the more extreme end of discrimination, racially motivated assault causes physical and emotional injury. Experiencing anxiety in anticipation of discrimination within specific settings due to past incidents may cause social isolation of both individuals and communities, which can subsequently contribute to mental disorders. In some cases, it may be that the discriminatory experiences themselves do not contribute directly to poorer health, but are mediated by other factors along the pathway—for example, if an individual experiences racial discrimination that prevents them from finding adequate employment, the resultant un- or underemployment may then contribute to poorer health outcomes.

Intersectionality theory posits that systems of oppression built on a range of factors including gender, sexuality, class, age and disability are both overlapping and mutually reinforcing; thus, individuals may simultaneously face multiple forms of discrimination [4,14,15]. Viruell-Fuentes contends that consideration of the intersection between racial or ethnic background and immigration status is essential for an in-depth understanding of migrant groups’ experiences of racism, discrimination and health [14]. Immigration status includes the amount of time spent in the country at both the individual and population level. While the evidence is contradictory, various studies have found that immigration status is associated with likeliness to report experiences of discrimination. Research in Australia has demonstrated that individuals who have arrived more recently report higher levels of discrimination [16], while studies in the United States have indicated that immigrants report more discrimination with increased time in the country [17]. Other studies have reported that immigration status may affect the strength of the association between discrimination and health [18].

In Australia, waves of migration from the Middle East and Asia began in the 1970s [19], with an increase in migration from the Middle East in the 1990s [20]. Migration from Africa also saw a significant increase in the later 1990s and first half of the 2000s [21]. Possibly due to their relatively short time in Australia, these populations tend to be poorly represented in the Australian health literature, necessitating further examination of factors that affect their health status, including racial discrimination. For example, while a search of the literature uncovered a number of studies examining the discrimination faced by African migrants in Australia [22,23], these were limited mainly to humanitarian entrants, notwithstanding the evidence demonstrating that a sizeable proportion of African-born migrants enter through skilled migration schemes [24,25].

The concepts of visibility, stigmatisation and discrimination are highly intertwined. Erving Goffman conceptualised stigma as an attribute that is devalued in a particular social context and highlighted race as a visibly identifiable characteristic that held particular social significance [26]. In the decades since, race as a signifier of a stigmatised condition has been explored through the lens of legal frameworks [27], social psychology [28] and health [29]. The initial step in perpetuating stigma is the perception of a characteristic as both tied to an individual and having social meaning. The characteristic is then linked to negative traits through the attribution of stereotypes, which are used to identify the individual as not belonging to the social norm. Discrimination is the subsequent behavioural expression of this rejection [30]. The visibility of the relevant characteristic is therefore particularly important in understanding how discrimination is perpetuated against a population group.

The Culturally and Linguistically Diverse (CALD) Experiences of Racism surveys were designed to assess racial and ethnic minorities’ self-reported experiences of interpersonal racism, their responses and reactions to these experiences and the association between these experiences and measures of psychological distress. The surveys were implemented as part of an associated anti-racism initiative in order to appropriately identify, prioritise and target specific settings for intervention.

While Aboriginal Australians are also vulnerable to racism [31,32], the experiences of CALD and Aboriginal communities are often considered separately in Australia, due to differing needs, contexts and histories. These differences are particularly salient when considering the impact of migration on discrimination experiences. A separate survey was therefore designed to assess experiences of racism in Aboriginal communities [31], and those findings are not included in this manuscript.

Through the CALD Experiences of Racism surveys, multiple dimensions of discriminatory experiences have been captured, including types of discriminatory experiences, where these experiences occurred and responses to these experiences. The surveys were designed to examine a dose–response relationship between experiences of racial discrimination and psychological distress, mediating effects of specific responses and differential impacts of experiencing particular types of racial discrimination or experiencing racial discrimination in particular settings. Interpersonal racism constitutes only one aspect of racism, each of which may be detrimental to health in varied and complex ways. This paper therefore presents a specific facet of racism’s effects on mental health, rather than a comprehensive overview of the impact of racism.

## Methods

The CALD Experiences of Racism surveys were developed to inform the Victorian Health Promotion Foundation’s Localities Embracing and Accepting Diversity (LEAD) program. The surveyed localities within the Australian state of Victoria were selected as areas that had a high level of racial and ethnic diversity where the local government recognised racism as a concern in the community and demonstrated both a capacity and commitment to addressing it. Selection was not due to particularly high levels of racism in comparison to other Victorian communities. The two rural and two metropolitan localities surveyed have been de-identified in order to protect the anonymity of the communities in question.

Participants in the CALD Experiences of Racism surveys were aged 18 years and older and lived within Rural Council 1 (n = 298), Metropolitan Council 1 (n = 335), Metropolitan Council 2 (n = 226) or Rural Council 2 (n = 280) for at least one year. ‘Councils’ refers to local governing bodies, and the areas governed by councils are local government areas (LGAs).

### Survey structure

Consultation was conducted in each area to ensure that the relevant Experiences of Racism survey was appropriate and accessible for each community. Translations were available in Arabic, Chinese (Traditional), Dari, Swahili, Tongan, Turkish, and Vietnamese. The surveys began with demographic questions including age, gender and education. Participants were asked to provide their racial, ethnic or cultural background, country of birth, parents’ countries of birth, length of time in Australia, language(s) other than English spoken at home and religion. Although a range of other factors such as existing chronic conditions and socio-economic status can also be associated with health outcomes, these were not measured in the surveys.

The surveys assessed interpersonal racism using a grid that had types of experiences listed on the left and settings listed across the top. Participants then indicated which type of experience had occurred and where it took place by marking the appropriate grid box. This method was based on a tool used previously with young Australians [33]. Experiences listed included racist name-calling or teasing; verbal abuse or offensive gestures; being told the participant does not belong in Australia; being left out or avoided; being treated as inferior or less intelligent; being ignored, treated with suspicion or treated rudely; having property vandalised; and physical abuse or the threat of physical abuse. Settings listed were: in a shop, store or mall; while doing sport, recreational or leisure activities; while seeking housing or in dealing with real estate personnel; in a bank or other financial institution; in dealings with local council; in dealings with other government agencies; at work, on the job or when looking for a job; at school, university or another educational setting; in public spaces (on the street, beach, park etc.); with the police, courts or jails; in hospitals, health centres, at the doctor’s office; on public transport; and ‘other’. Experiences and settings were based on the Scanlon Foundation’s Mapping Social Cohesion Survey [34], the Everyday Discrimination Scale [35], the Racism and Life Experiences Scale [36], the Experiences of Discrimination scale [37], the Schedule of Racist Events [38] and the Measure of Indigenous Racism Experiences (MIRE) [39]. The tool used to assess experiences of racism is illustrated in Table 1.

**Table 1:** **Tool to assess experiences of racism**

	**Where?**												
Have you ever…	in a shop, store or mall	while doing sport, recreational or leisure activities	while seeking, housing or in dealing with real estate personnel	in a bank or other financial institution	in dealings with your local Council	in dealings with other government agencies	at work, on the job or when looking for a job	at school, university or another educational setting	in public spaces (on the street, beach, park etc.)	with the police, courts or jails	in hospitals, health, centres, at the doctor’s office	on public transport	other
been a target of racist names, jokes or teasing or heard comments that rely on stereotypes of your racial, ethic, culture or religious group?													
been sworn at, verbally abused or had someone make offensive gestures because of your race, ethnicity, culture or religion?													
had someone suggest you do not belong in Australia, that you should ‘go home’ or get out’ and so on?													
felt left out or avoided because of your race, ethnicity, culture or religion?													
had someone treat you as less intelligent, or inferior, because of your race, ethnicity, culture or religion?													
been ignored, treated with suspicion or treated rudely because of your race, ethnicity, culture or religion?													
had your property vandalised because of your race, ethnicity, culture or religion?													
had someone spit or throw something at you or hit you or threaten to hit you because of your race, ethnicity, culture or religion?													

Participants who responded that they had experienced at least one discriminatory incident over the past twelve months received a series of questions asking for details about their most recent experience, including how stressful the incident was and actions that the participant took in relation to the incident. Responses to racial discrimination were based on items in the Experiences of Discrimination Scale [37], the Schedule of Racist Events [38] and the MIRE [39].

Mental health was assessed through the inclusion of the Kessler 6 (K6) scale. The scale is a quantifier of non-specific psychological distress, which was derived from the Kessler Psychological Distress Scale (K10) as a simple measure of psychological distress. The K6 has demonstrated excellent internal consistency and reliability as well as consistency across major socio-demographic sub-samples [40]. The K6 involves six questions about emotional states, each with a five-level response scale. The measure can be used as a brief screen to identify levels of distress. The K6 can be given to participants to complete, or alternatively the questions can be read to the participant by the administrator.

The K6 is scored using the sum of answer responses, where responses of ‘None of the time’ are given a score of one to ‘All of the time’ yielding a score of five. Thus the range of responses is 6–30. Using the K6, respondents are classified as being at low, moderate, high or very high psychological distress, which is in turn correlated with the risk of having a mental disorder. Low scores indicate low levels of psychological distress and high scores indicate high levels of psychological distress. There are a number of different categories and groupings used for analysis of the K10 and K6 scores. However, in most Australian Bureau of Statistics (ABS) and other Australian surveys, the data is presented according to four categories (low, moderate, high and very high), with a very high score of psychological distress possibly indicating a need for professional help [41]. K6 scores of 19 to 30 indicate high or very high psychological distress [41].

### Survey administration

Community workers were recruited to administer the surveys in each area (Metropolitan Council 1 = 9; Metropolitan Council 2 = 9; Rural Council 2 = 12), excluding the community in Rural Council 1. The recruitment process included a consultation phase with relevant stakeholder groups in each council. This process was used to identify the most appropriate way of recruiting community workers and to develop data governance protocols. Community workers were trained in ethical research practices and survey administration by the LEAD evaluation team and supported throughout the data collection period through frequent contact with evaluation team members. Community workers distributed surveys through their personal and professional contacts as well as through local community events and functions. Surveys were administered face-to-face in group or individual sessions. The community workers who administered the surveys recorded both participants and people who were invited but declined to participate. The reasons provided for declining to participate were recorded. Community workers also participated in a follow up session for feedback and debriefing. Participants received a $20 supermarket gift voucher after completing the surveys. Surveys were conducted in September 2010 in Metropolitan Council 1, between November 2010 and May 2011 in Metropolitan Council 2 and between March and May 2011 in Rural Council 2.

In Rural Council 1, community members were invited to complete the survey and have dinner during an event in July 2010 where 254 community members participated. Translated surveys were available and LEAD staff assisted community members to complete the survey where required. A further 44 community members were surveyed between July and December 2010 by LEAD staff to bring the total number of participants in Rural Council 1 to 298. This process was developed under the advice and in consultation with local service providers and council.

### Data analysis

SPSS Statistics 19 was used to analyse these data. Participants’ experiences of racial discrimination were divided into None, Low (1–5 experiences), Medium (6–8) and High (9+) frequency categories. These cut-off points were selected so approximately one third of people who experienced racism were in each category. Chi-square analysis was used to assess demographic differences between people with different frequency levels. Pearson’s correlation was used to assess the relationship between exposure and scores on the K6 univariately. Logistic regression was used to assess the relationship between the participants’ experiences of discrimination and position above or below the threshold for high or very high psychological distress on the K6 scale. Logistic regression was also used to assess the relationship between the type of discriminatory incident and being above or below the threshold for high or very high psychological distress. Logistic regression was conducted to examine the effects of experiencing discrimination in particular settings on being above or below the threshold for high or very high psychological distress as well as the role of response strategies on stress associated with the most recent discriminatory incident. Stress was coded into two categories (Not at all/a little/somewhat stressful and very/extremely stressful). All models controlled for age, gender, education and LGA as potential confounding factors. Visibility status was determined primarily by country of birth. For respondents who were born in Australia, New Zealand or northern European countries, visibility status was determined by self-reported racial, ethnic or cultural background. Visibility was coded as Low (European background), Medium (Pacific Islands, Latin America and the Caribbean), High (East and South Asia and Middle East) and Very high (Sub-Saharan Africa). Visibility status clusters were based on the composition of immigration waves into Australia from the 1990s onward. Previous studies have used similar methods to examine differential experiences of discrimination between migrant communities in Australia [42]. As some participants did not complete every item, valid percents are reported for all frequencies, with missing data removed.

### Ethics

Ethical approval to conduct the study was granted by the Melbourne School of Population and Global Health Human Ethics Sub-Committee (MSPGH-HESC) on 27 January 2010 (ID number 0932878).

## Results

A total of 1139 people participated in the CALD Experiences of Racism surveys. The response rate across all LGAs was 96%.

### Demographic data

Demographic data for participants are presented in Table 2. Participants were fairly evenly distributed across the four LGAs, although Metropolitan Council 1 and Rural Council 1 represented slightly more participants. The majority of participants were women. The mean age of the sample was 36 years and a majority of the sample was highly visible. More than one third of participants held either tertiary, trade or TAFE qualifications. The most frequently represented religion was Islam, followed by Christianity.

**Table 2:** Demographic data

		**n** ^**a**^	**%**
*LGA*	Rural Council 1	298	26.2
	Metropolitan Council 1	335	29.4
	Metropolitan Council 2	226	19.8
	Rural Council 2	280	24.6
*Gender*	Male	541	47.5
	Female	580	50.9
*Age*	18-24	257	22.6
	25-34	246	21.6
	35-44	217	19.1
	45-54	155	13.6
	55-64	75	6.6
	65+	39	3.4
*Visibility*	Low	88	7.7
	Medium	146	12.8
	High	628	55.1
	Very high	262	23.0
*Education*	Tertiary qualifications	271	23.8
	Trade or TAFE	141	12.4
	Higher School Certificate	267	23.4
	School certificate	109	9.6
	Primary school	79	6.9
	Other	119	10.4
*Religion*	Buddhism	44	3.9
	Christianity	351	30.8
	Hinduism	69	6.1
	Islam	435	38.2
	Sikhism	53	4.7
	Other	8	0.7
	None	50	4.4
*Country of birth*	Australia/New Zealand	69	5.8
	Middle East	306	25.6
	Africa	259	21.7
	East Asia	166	13.9
	South Asia	122	10.2
	Pacific Islands	116	9.7
	Europe	74	6.2
	Americas	4	0.4
*Level of experiences*	None	418	36.7
	Low	264	23.2
	Medium	251	22.0
	High	206	18.1
*Time in Australia*	0-5	366	37.5
*(years)*	5-10	244	25.1
	10-15	111	11.4
	15-20	89	9.1
	20+	165	16.7

^a^N may not add to 1139 due to missing values; percentages may not add to 100 per cent due to rounding.

### Experiences of racism

Nearly two thirds of participants reported at least one discriminatory experience in the preceding twelve months, with 23% reporting between one and five experiences, 22% reporting between six and eight experiences and 18% of all respondents reporting nine or more experiences. No experiences were reported by 37% of respondents.

**Figure 1:**
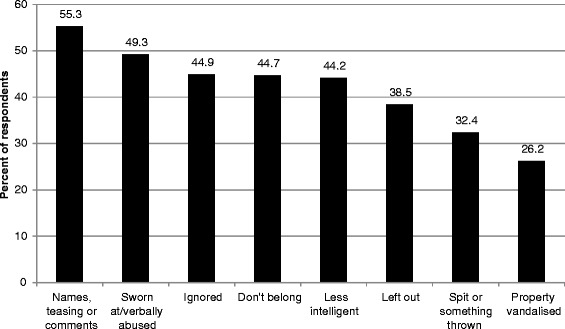
Experiences of racism for Victorians from racial/ethnic minority backgrounds.

The frequency of experiences roughly corresponded inversely with their intensity, with racist verbal experiences being reported more frequently than physical violence or the destruction of property. The most frequent experience reported was being a target of racist names, jokes or teasing, or hearing comments that rely on stereotypes of the participant’s racial, ethnic cultural or religious group. This experience was reported by 55% of participants. Having property vandalised was reported by more than one quarter of participants (Figure 1).

**Figure 2:**
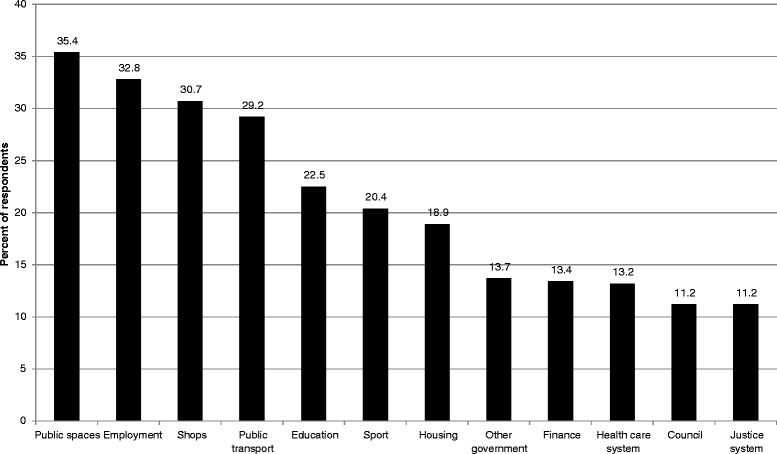
Settings where Victorians from racial/ethnic minority backgrounds experienced racism.

Respondents indicated that discrimination was most commonly reported in public spaces, with 35% indicating that they had experienced a discriminatory incident in public spaces in the prior twelve months, followed by employment (33%) and a further 30% each experiencing incidents in shops and public transport (Figure 2). Data were not collected on perpetrators in specific settings. Therefore, it is not known whether the racist behaviours in settings such as health care, local council, or justice settings were initiated by staff, clients or others.

People who had experienced discrimination used a range of methods to respond to these incidents. Twelve responses were listed, plus an ‘other’ category, with participants able to choose however many applied to their most recent experience. With regards to respondents’ most recent experiences, the two most common responses were to ignore it/pretend it didn’t happen and to accept it as a fact of life/put up with it (45% and 27% respectively of those who reported at least one experience of discrimination).

Age, education, religion, gender and rurality were all significantly associated with differences in the frequency of experiencing racial discrimination (Table 3). People living in metropolitan areas were more likely to experience discrimination than people living in rural areas and when they experienced discrimination they were more likely to report high levels of experiences. There were no differences between genders according to rurality. Overall, women were less likely than men to report experiencing racial discrimination in the last twelve months and when they did report such experiences, women were less likely to be in the high frequency category (Table 4). The proportion of people who were in the high frequency category decreased with age. This effect was relatively consistent across genders. University-educated people were also more likely to report higher levels of experiencing racial discrimination than non-university educated people.

**Table 3:** **Experiences of racism by demographic characteristics**

**Setting**	***χ*** ^**2**^	**df**	**p**
Age	60.9	12	<0.01
Education	54.09	9	<0.01
Religion	49.45	15	<0.01
Gender	18.42	3	<0.01
Rurality	33.40	3	<0.01

**Table 4:** **Experiences of racism by gender**

**Setting**	**Men**		**Women**	
	**χ2**	**p**	**χ2**	**p**
Age	37.16	<0.01	27.02	0.01
Education	60.61	<0.01	15.90	0.07
Religion	22.22	0.1	37.19	<0.01
Rurality	20.19	<0.01	13.09	<0.01

**Figure 3:**
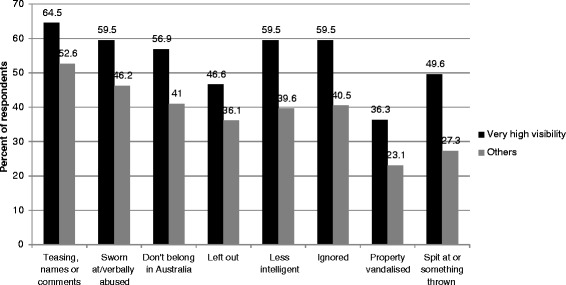
Visibility and experiences of racism.

**Figure 4:**
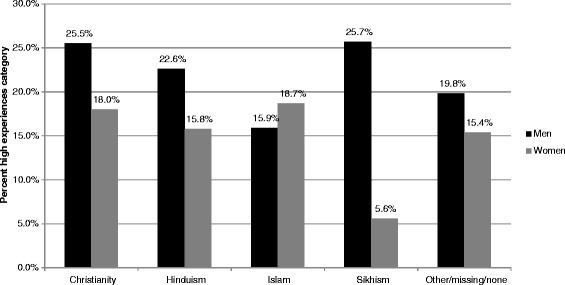
High experiences of racism by religion and gender.

People with very high visibility reported higher levels of all types of experiences than others (Figure 3). While there were very few differences between genders associated with visibility, across religious categories Muslims were the only group within which women were more likely to experience high levels of racial discrimination than men. The largest gender difference among religious categories was seen in Sikhism, with men much more likely to experience high levels of racial discrimination than women (Figure 4).

### Mental health and racism

Scores of 19 to 30 on the K6 scale are indicative of high or very high psychological distress [41]. The mean K6 score for the sample was 13.5, with 17.5% scoring over the threshold for high or very high psychological distress.

More frequent experiences of racial discrimination were related to increased psychological distress as indicated by a higher score on the K6 (r = 0.37, p = 0.01). People who experienced medium and high levels of discrimination were significantly more likely to be above the threshold for high or very high psychological distress compared to people who had no experiences of racism (Table 5).

**Table 5:** **Experiences of racism and odds of being above the threshold for high or very high psychological distress on the K6**

**Experiences**	**Odds of being above threshold for high or very high psychological distress**		**% above threshold for high or very high psychological distress**
	**OR 95% CI** ^**a**^	**p**	
None	Reference		7.6
Low (1–5)	1.55, (0.79-3.04)	0.2	10.6
Medium (6–8)	3.49, (1.82-6.7)	<0.01	20.3
High (9+)	14.93, (8.23-27.08)	0.01	42.3

^a^Odds ratio adjusted for age, gender, education and LGA.

The type of discrimination experienced was not significantly associated with being above the threshold for high or extremely high psychological distress on the K6. However, experiencing discrimination in shops and in employment and government settings was significantly associated with being above the threshold for high or very high psychological distress on the K6 (Table 6).

**Table 6:** **Settings of racist experiences and odds of being above the threshold for high or very high psychological distress on the K6**

**Setting**	**B**	**SE B**	**OR**	**95%** **CI**	**p**
Ina shop, store or mall	0.561	0.246	1.753	(1.082-2.839)	0.023
While doing sport, recreational or leisure activities	0.461	0.256	1.586	(0.960-2.619)	0.072
While seeking housing or in dealing with real estate personnel	0.069	0.275	1.071	(0.625-1.834)	0.803
In a bank or other financial institution	0.190	0.312	1.209	(0.656-2.227)	0.543
In dealings with your local Council	0.384	0.326	1.469	(0.775-2.783)	0.238
In dealings with other government agencies	0.622	0.310	1.862	(1.014-3.418)	0.045
At work, on the job or when looking for a job	0.531	0.238	1.701	(1.066-2.714)	0.026
At school, university or another educational setting	−0.119	0.253	0.888	(0.541-1.456)	0.637
In public spaces (on the street, beach, park etc.)	−0.059	0.245	0.943	(0.583-1.524)	0.809
With the police, courts or jails	0.491	0.310	1.633	(0.890-2.996)	0.113
Hospitals or health services	−0.463	0.313	0.629	(0.341-1.162)	0.139
Public transport	0.388	0.253	1.474	(0.898-2.421)	0.125
Other	0.006	0.292	1.006	(0.567-1.784)	0.983

‘Ignoring it or pretending it didn’t happen’, a response utilised by almost half of participants, was the only strategy associated with decreased odds of finding the last incident very stressful or extremely stressful (Table 7). No other response strategies were significantly associated with stress resulting from participants’ last racist experience.

**Table 7:** **Responses to last experience and finding the experience stressful or very stressful**

**Response strategies for most recent racist experience**	**B**	**SE B**	**OR**	**95%** **CI**	**p**
Ignored it or pretended it didn’t happen	−0.661	0.203	0.516	(0.347-0.768)	0.001
Accepted it as a fact of life or put up with it	0.129	0.223	1.138	(0.735-1.762)	0.563
Wanted to face up to the person who did this to you but didn’t	0.337	0.247	1.401	(0.864-2.272)	0.172
Tried to reason with the person who did this to you	0.332	0.302	1.393	(0.771-2.517)	0.272
Used humour or ridiculed the person who did this to you	−0.611	0.374	0.543	(0.261-1.129)	0.102
Sought or accepted help from others who saw/heard it happen	0.654	0.507	1.924	(0.713-5.194)	0.197
Got into a verbal confrontation with the person who did this to you	−0.080	0.286	0.923	(0.526-1.618)	0.779
Wrote, drew, sang or painted about the experience	−0.357	0.684	0.699	(0.183-2.674)	0.601
Talked to someone about the experience	0.324	0.232	1.383	(0.878-2.180)	0.162
Made a complaint to an organisation or agency	0.208	0.412	1.231	(0.549-2.763)	0.614
Reported to the police or took legal action	0.214	0.410	1.239	(0.554-2.770)	0.601
Tried to change the way you are or things you did to avoid it in the future	0.202	0.292	1.224	(0.691-2.171)	0.488
Other coping strategy	0.371	0.569	1.449	(0.475-4.419)	0.514

## Discussion

Supporting a few existing Australian studies [43,44], the current research demonstrates that for many Australians from racial and ethnic minority backgrounds, experiencing racial discrimination is a regular occurrence associated with increased psychological distress and risk of mental illness. In addition to mental health implications, some of the experiences reported by respondents also have negative ramifications for physical health, including being spat on or physically assaulted. The study results support the growing body of literature that links experiencing racial discrimination to negative health outcomes [8,9,11,45] and highlight the need for interventions to protect the mental and physical health of racial and ethnic minority communities through addressing racial discrimination [1,46,47].

The largest proportion of respondents had been in the country for fewer than six years and more than half of respondents had been in Australia for ten years or fewer. The current results illustrate the impact of social visibility on experiences of racial discrimination and are consistent with evidence indicating that ethnic minority communities that have spent longer in Australia are more accepted by the general community than those that arrived more recently [48]. Visibility in general Australian society across multiple axes was associated with higher rates of experiencing racial discrimination. Individuals who were highly visible as determined by country of birth and/or self-reported racial, ethnic or cultural backgrounds were subject to higher levels of all types of racist experiences than others. This is congruent with research demonstrating high levels of employment discrimination based on visibility in Australia and higher levels of perceived discrimination in public spaces as well as lower life, employment and financial satisfaction for highly visible migrants in Australia in comparison to less visible migrants [42,49]. The international literature also supports the general trend of more visible migrant groups experiencing higher levels of racism than less visible groups [50-52].

Increased visibility is therefore also likely to account for Muslim women reporting higher levels of racism than Muslim men (i.e., because many Muslim women are identifiable due to wearing hijabs, niqabs or burqas) and the reverse trend for Sikhs (i.e., because Sikh men are identifiable by their turbans). Research in Australia indicates that some employers view religious dress, prayer or practices as legitimate reasons to reject a job applicant, particularly for public-facing positions [49]. Internationally, Muslim veiling practices have been constructed as a threat to ‘the British way of life’ [53] and wearing the turban has been placed in opposition to ‘Canadian traditions’ [54].

While participants were not asked about visible religious markers (e.g., hijab for Muslims or turbans for Sikhs), it is probable that the high levels of experiences in these groups are more closely associated with visible markers of non-Christian religions, rather than self-identification alone. The CALD Experiences of Racism surveys are unusual in examining both religious and racial/ethnic background in relation to discriminatory experiences. However, this approach is supported by the link between religious and ethnic identities, the racialisation of religion and discriminatory targeting of religious minorities [6,7,55]. Possibly due to the lack of studies incorporating religion into their analyses, our findings relating to the effect of gender on experiences of discrimination for Sikhs and Muslims are novel to the best of our knowledge.

Experiencing racial discrimination was associated with worse mental health, with the odds of being above the threshold for high or very high psychological distress significantly higher for people with medium and high levels of experiences of racism compared to people who had no experiences of racism. Mental health impacts were associated with the volume of racist experiences but not the type of racist incident. This finding suggests that all types of racism can have detrimental impacts on mental health.

Experiencing racial discrimination in some specific settings was also shown to be more strongly associated with being above the threshold for high or very high psychological distress. Experiencing discriminatory incidents in shops and in employment and government settings was significantly associated with being above the threshold for high or very high psychological distress on the K6. While studies examining differential mental health effects of experiencing racial discrimination in different settings are limited, there is evidence indicating that racism in some settings can be more harmful than racism in others. For example, recent research highlights that experiencing racism in health settings may have a stronger negative impact on mental health than experiencing racism in other settings [56]. The association between reported racial discrimination and adolescent smoking has been found to be influenced both by gender and by setting, with the likelihood of smoking among girls, but not boys, being associated with discrimination in school, work, and neighbourhood settings [57]. Liebkind and Jasinkaja-Lahti found that ‘Everyday Racism’, ‘Discrimination in Services’ and ‘Discrimination at Work’ each had differential impacts on psychological stress among different ethnic/racial groups and between genders [51]. However, each of these factors was constructed from items relating to a range of situations and settings.

The higher levels of psychological distress associated with racial discrimination in shops are particularly concerning as this is one of the five most frequently reported settings. In addition to presenting a barrier to accessing everyday goods and resources, experiencing discrimination in public places such as shops may signal social isolation and exclusion from participants’ local areas, particularly in combination with the high proportion of discriminatory incidents reported in general public spaces in this and other studies [31,58,59]. Social exclusion and isolation are important determinants of mental health in themselves, contributing to psychological distress [60-63].

Experiencing racism within government settings may prevent people from racial and ethnic minority backgrounds from being adequately informed about or able to access available government services or support they may be entitled to [64]. Racism within government settings is also likely to interfere with racial and ethnic minority communities’ right to civic and social participation which can then prevent adequate representation of people from racial and ethnic minority backgrounds in forming governmental policies and programs.

Experiencing racial discrimination in employment settings within the previous twelve months was shown to be more strongly associated with being above the threshold for high or very high psychological distress. In addition, both employment and education were within the top five settings for experiencing discrimination. The high levels of racial discrimination reported in these settings may represent a particularly pernicious threat to equity in opportunities and resources for Australians from minority racial and ethnic backgrounds. Discrimination in employment and education may result in a reduction in life chances for people from racial and ethnic minority backgrounds, which has serious long-term implications for their mental health and wellbeing. Mental health inequality is at least partly linked to income inequality, which is in turn associated with differential employment and education outcomes [65-67]. The finding that employment discrimination is relatively common is consistent with other Australian literature. A study of black African nurses identified racist stereotypes of this sample to include perceptions of incompetence, expressed in the form of pervasive, subtle and implicit remarks that undermined their professional status or skillset [68]. For people from Asia and the Middle East, employment discrimination has been identified as particularly pertinent to their experiences, including for entrants under Australian skilled migration programs [69]. One study found that identical curriculum vitae (CVs) with Chinese or Middle Eastern names received significantly fewer interviews than CVs with Anglo or Italian names [70]. The authors of this study found some evidence that discrimination against hiring Chinese or Middle Eastern applicants was more pronounced for jobs requiring higher degrees of customer interaction [70].

The use of coping mechanisms has been shown to modify the mental health effects of experiencing racial discrimination—either exacerbating negative health outcomes or partially countering them—although these findings are somewhat contradictory [10,71]. In the current study, ‘Ignoring it or pretending it didn’t happen’ was the only strategy that was associated with decreased odds of finding the last incident very stressful or extremely stressful. However, this finding contradicts some existing studies which suggest that this coping response exacerbates the mental health ill-effects of racism, rather than being useful in mediating negative health effects [8,72,73]. Such contradictory findings may result from two distinct uses of this coping strategy. The first is as a form of ‘avoidance coping’, ‘in which the individual does not directly address the problem and engages in activities that lead to withdrawal from day-to-day activities’ [73]. While this form of coping is considered to be maladaptive, an alternate use of this strategy as ‘a conscious decision to ignore racist incidents, placing the responsibility…squarely on the shoulders of the perpetrators’ can be empowering in some situations [4,71]. However, ignoring racism is not always a viable option, given that some of the actions reported in the surveys are not only harmful but illegal, such as vandalising property, physical violence or the threat of physical violence [71].

Given that a single response out of the twelve possible strategies was associated with finding the most recent incident very stressful or extremely stressful, the study results support the conclusion that interventions designed to prevent the occurrence of racism have more potential to increase mental health in racial and ethnic minority communities than interventions that work with individuals on responses to experiences of racism.

### Limitations

While the study focused on participants’ perceptions of experiencing racism and did not attempt to discern perpetrators’ motives, many of the behaviours reported are quite unambiguous, including being spat at or experiencing physical or verbal abuse. However, it is important to note that the international literature clearly indicates that racism tends to be under-reported rather than over-reported [74,75] and that people may act in ways that are discriminatory or racist without malice or awareness [76-79]. Regardless of the intent of the perpetrators, the results of the study clearly demonstrate that the perception of experiencing racism has a negative health impact.

Both the K6 and K10 have been tested and shown validity and reliability across a number of populations in various studies, including in such diverse contexts as Japan [80] and Burkina Faso [81]. The K10 has been used by the ABS across a number of population-level surveys [82]. An abbreviated form of the K10, the K5, is also used by the ABS in Aboriginal Australian populations [83]. The scope of prior testing of the Kessler scales and its use in Australian populations supports its use in the current study. However, the K6 has not specifically been tested for validity in racial and ethnic minority populations in Australia.

There is the possibility that use of community workers and interpreters may have led to research bias. To avoid this, training in ethical research methods and survey administration, including standardisation of data collection, was conducted with all community workers. The importance of standardisation was also reiterated as part of the ongoing support provided to community workers during data collection.

The purpose of this study was to explore the associations between experiences of racism and mental health outcomes, rather than examining the prevalence of racism within or between the four localities. In addition, data to determine whether the psychological distress profile of this sample is representative of the wider Victorian CALD population were not available. Recruitment methods were not necessarily intended to result in a representative sample. However, the validity of the relationship observed between experiences of racism and mental health status in this study is unlikely to be biased by the non-representative sampling techniques used.

The data is cross-sectional, so there is the potential for reverse causation; that is, people above the threshold for high or very high psychological distress may be more exposed to racism. There is, however, a wide body of evidence suggesting an association between racism and ill-health in longitudinal studies, with very little research suggesting reverse causation [8,84,85].

## Conclusions

The results of this study highlight the pathways between exposure to racism and poorer mental health outcomes and life chances for Australians from racial and ethnic minority backgrounds. While poorer mental health was associated with the volume, rather than the type of racist incident experienced, the impact of experiencing discrimination in some settings was shown to be particularly associated with high or very high psychological distress.

Although evidence of the health benefits of anti-racism interventions has only recently emerged [86], study findings suggest that preventing racial discrimination will be a more constructive approach to protecting the health of racial and ethnic minority communities than relying on the use of appropriate response mechanisms after a racist incident has occurred. A strong understanding of the patterns of racism experienced and ways in which racism influences health is therefore crucial for the development and implementation of relevant intervention strategies and allows more effective targeting of efforts to improve the health of affected populations [9]. In particular, the various ways that racism can lead to poorer health outcomes indicate the need for multi-level, multi-setting and multi-strategy interventions [1].

For example, employment and education are two of the settings where legislation exists in most countries to prohibit discrimination and legal recourse is available for people who have experienced discrimination [87]. Nevertheless, both employment and education were within the top five settings in which discrimination was most commonly reported, indicating that current frameworks and resources may be inadequate to prevent discrimination from occurring in these settings. More work may be needed to support employers and educators to comply with existing anti-discrimination legislation. Alternatively, strategies that promote social cohesion within a community may support social norms that curtail the expression of racism in public spaces and minimise the exclusion of racial and ethnic minority communities from public life.
